# The influence of intestinal parasites on *Plasmodium vivax*-specific antibody responses to MSP-1_19_ and AMA-1 in rural populations of the Brazilian Amazon

**DOI:** 10.1186/s12936-015-0978-7

**Published:** 2015-11-06

**Authors:** Juan Camilo Sánchez-Arcila, Marcelle Marcolino de França, Virginia Araujo Pereira, Mariana Pinheiro Alves Vasconcelos, Antonio Têva, Daiana de Souza Perce-da-Silva, Joffre Rezende Neto, Cesarino Junior Lima Aprígio, Josue da Costa Lima-Junior, Mauricio Martins Rodrigues, Irene Silva Soares, Dalma Maria Banic, Joseli Oliveira-Ferreira

**Affiliations:** Laboratorio de Imunoparasitologia, Instituto Oswaldo Cruz, Fundação Oswaldo Cruz, Av. Brasil 4365, Manguinhos, Rio de Janeiro, Brazil; Instituto de Infectologia Emílio Ribas, São Paulo, Brazil; Laboratório de Imunodiagnóstico, Departamento de Ciências Biológicas, Escola Nacional de Saúde Pública/Fiocruz, Rio de Janeiro, Brazil; Laboratório de Simulídeos e Oncocercose, Instituto Oswaldo Cruz, Fundação Oswaldo Cruz, Rio de Janeiro, Brazil; Intituto de Gastroenterologia de Goiânia, Goiânia, Goiás Brazil; Agencia de Vigilância em Saúde da Secretaria de Estado da Saúde - AGEVISA, Rondonia, Brazil; Centro de Terapia Celular e Molecular (CTCMol), Universidade Federal de São Paulo, Escola Paulista de Medicina, São Paulo, Brazil; Departamento de Análises Clínicas e Toxicológicas, Faculdadede Ciências Farmacêuticas, Universidade de São Paulo, São Paulo, Brazil

**Keywords:** Malaria, Intestinal parasites, Co-infection, *Plasmodium vivax*, AMA-1, MSP-1_19_

## Abstract

**Background:**

Polyparasitism is a common condition in humans but its impact on the host immune system and clinical diseases is still poorly understood. There are few studies of the prevalence and the effect of malaria-intestinal parasite co-infections in the immune response to malaria vaccine candidates. The present study determines whether the presence of malaria and intestinal parasites co-infection is associated with impaired IgG responses to *Plasmodium vivax* AMA-1 and MSP-1_19_ in a rural population of the Brazilian Amazon.

**Methods:**

A cross-sectional survey was performed in a rural area of Rondonia State and 279 individuals were included in the present study. At recruitment, whole blood was collected and *Plasmodium* and intestinal parasites were detected by microscopy and molecular tests. Blood cell count and haemoglobin were also tested and antibody response specific to *P. vivax* AMA-1 and MSP-1_19_ was measured in plasma by ELISA. The participants were grouped according to their infection status: singly infected with *Plasmodium* (M); co-infected with *Plasmodium* and intestinal parasites (CI); singly infected with intestinal parasites (IP) and negative (N) for both malaria and intestinal parasites.

**Results:**

The prevalence of intestinal parasites was significantly higher in individuals with malaria and protozoan infections were more prevalent. IgG antibodies to PvAMA-1 and/or PvMSP-1_19_ were detected in 74 % of the population. The prevalence of specific IgG was similar for both proteins in all four groups and among the groups the lowest prevalence was in IP group. The cytophilic sub-classes IgG1 and IgG3 were predominant in all groups for PvAMA-1 and IgG1, IgG3 and IgG4 for PvMSP-1_19_. In the case of non-cytophilic antibodies to PvAMA-1, IgG2 was significantly higher in IP and N group when compared to M and CI while IgG4 was higher in IP group.

**Conclusions:**

The presence of intestinal parasites, mainly protozoans, in malaria co-infected individuals does not seem to alter the antibody immune responses to *P. vivax* AMA-1 and MSP-1_19_. However, IgG response to both AMA1 and MSP1 were lower in individuals with intestinal parasites.

## Background

It is well known that polyparasitism is a common condition in humans, however, little is known about the interaction between parasites and its impact on the host immune system and clinical diseases [1]. Malaria and intestinal parasitic infections are distributed throughout the world and are highly prevalent in humid and warm environments in the tropics. The World Health Organization estimates that 3.3 billion people, almost half the world’s population, are at risk of contracting malaria and approximately 3.5 billion people are affected by intestinal protozoa and/or helminths [2]. Thus, protozoa of the genus *Plasmodium*, etiological agents of malaria, and many species of intestinal parasites (protozoa and helminths) share the same geographic distribution area and both types of parasites can infect the same population of hosts.

The implication of concomitant infection in humans has been evaluated mainly regarding the effects of intestinal helminth infections on falciparum malaria, obtaining conflicting results. While *Ascaris lumbricoides* infection may protect against cerebral malaria [3, 4] and *Schistosoma haematobium* has a protective effect on the density of the *Plasmodium falciparum* infection, [5, 6] children carrying intestinal helminth infections including *Ascaris lumbricoides* were more susceptive to either *P. falciparum* infection or acute malaria attacks [7–9]. In other studies, co-infections can make no difference [10–13]. In rodent models of co-infection, schistosome and plasmodia infections are affected at the immunological level [14–17]. In humans, studies demonstrating an effect of helminths on vaccine-induced immune responses against influenza, cholera and tetanus have been described [18, 19]. So far, little information is available about whether and how co-infections of helminths and malaria parasites can affect specific immune response to malaria parasites and vaccine candidates [20–26]. In some epidemiological studies schistosomiasis co-infection favors anti-malarial protective antibody responses [21, 25] while in others no significant association between schistosome-specific and *Plasmodium*-specific antibody responses was observed [22, 23]. Similarly, systemic cytokine levels rose with age as well as with infection and exposure to schistosome or had no effect [22, 26].

The effects of helminths on falciparum malaria in humans remain uncertain and few data are available about the interaction between intestinal parasites and *Plasmodium vivax* [27]. In Brazil, *P. vivax* is the most prevalent malaria species corresponding to 83.7 % of the 134,907 cases registered in 2014 and it is concentrated in the Amazon region where intestinal parasites infections are prevalent [28, 29].

AMA-1 is expressed on merozoites and sporozoites as a type I integral membrane protein and MSP-1 is expressed abundantly on the merozoite surface and synthesized as a 195-kDa protein and sequentially processed into a cysteine-rich 19-kDa fragment (MSP-1_19_) [30, 31]. The proteins MSP-1 and AMA-1 are promising vaccine candidates for both *P. falciparum* and *P. vivax* and they are involved in erythrocyte invasion [32, 33].

Therefore, the aim of the study was to determine the prevalence of co-infection of malaria and intestinal parasites and whether the presence of co-infection was associated with impaired IgG responses against *P. vivax* proteins, apical membrane antigen-1 (AMA-1) and merozoite surface protein (MSP-1_19_) in individuals co-infected with *Plasmodium* and intestinal parasites and in individuals with single infections.

## Methods

### Study population

The individuals who took part in this study were part of a previous study investigating the effect of intestinal parasites on the circulating levels of cytokines and inflammatory markers [20]. The study area and population were also described in detail in this study. Briefly, a cross-sectional survey was conducted in a rural settlement community of Porto Velho, municipality of Rondonia State, and Brazilian Amazon. Only individuals that lived in the area and provided a blood sample and stool samples were included in the study (279 participants).

### Ethical consideration

Ethical approval was given by the Fundação Oswaldo Cruz Ethical Committee (CEP/FIOCRUZ, 492/08). Informed and written consents were obtained from all participants. For all eligible participants a clinical examination was performed. Donors positive for *P. vivax* and/or *P. falciparum* at the time of blood collection were subsequently treated using the chemotherapeutic regimen recommended by the Brazilian Ministry of Health. Participants positive for intestinal parasites were also treated.

### Sample collection and diagnosis

After written informed consent and an epidemiological survey from all adult donors or from parents of donors in the case of minors, blood samples were collected by venipuncture for serological assay and a thick and thin blood smear was prepared for microscopic detection of *Plasmodium*. Parasitaemia was expressed as the number of parasites/µL of blood in the thick blood smear. To confirm the parasitological diagnoses, molecular analyses of all samples using primers specific for genus (*Plasmodium* sp.) and species (*P. falciparum* and *P. vivax*) were done. PCR amplification and detection and the PCR primers used have been previously described [34]. Subjects positive in the thick blood smear and/or PCR were considered positive for malaria infection. Blood cell counts, including haematologic indices, were done using an automatic haematology analyzer (Pentra, Horiba Medical, Montpellier, France). The individuals were considered anaemic when their haemoglobin levels were ≤13 g/dL blood in males and ≤12 g/dL blood in females.

### Collection and examination of stool samples

For parasitological examinations, participants were requested to provide a morning faecal sample and a labelled screw-capped plastic container was provided. A single stool sample was collected from each subject on the following day and samples were screened for intestinal parasites and examined at the same day by direct wet mount microscopic and concentration techniques by a technician with expertise in intestinal parasites identification.

### Specific antibody for *Plasmodium vivax* antigen-specific IgG antibody and sub-classes in plasma samples

Specific IgG antibodies to PvMSP-1_19_ and PvAMA-1 in plasma were determined by enzyme-linked immunosorbent assays (ELISA). The expression and purification of the recombinant proteins were performed as previously described [35, 36]. The recombinant proteins were diluted in phosphate-buffered saline (PBS) pH 7.2 to a concentration of 2 μg/ml. High-binding ELISA plates (Nunc/Maxicorp) were coated with 100 μL of recombinant proteins and incubated overnight at 4 °C. Plates were washed four times with washing buffer, PBS-0.05 % Tween 20 (PBS-T) and were then blocked with blocking buffer (PBS-T containing 5 % low-fat milk) for 2 h at 37 °C. Individual plasma sample were diluted 1:100 in blocking buffer, 100 µl were added in duplicate to the respective wells and incubated for 1 h at 37 °C. After four washes with PBS-T, bound antibodies were detected with peroxidase-conjugated goat antihuman IgG (Sigma, St Louis, MO, USA) followed by *o*-phenylenediamine and hydrogen peroxide. The absorbance was read at 492 nm using an ELISA reader (Spectramax 250, Molecular Devices, Sunnyvale, CA, USA). The results for total IgG were expressed as reactivity indices (RI), which were calculated by dividing the mean optical density (OD) values of tested samples by the mean OD values plus three standard deviations (SD) of 24 non-exposed control individuals living in non-endemic areas of malaria (cut-off: PvAMA-1 = 0.1881, PvMSP-1_19_ = 0.1915). As positive controls, five plasma samples from exposed native individuals with high antibodies OD levels for both proteins were used. Subjects were considered as positive to the corresponding antigen if the RI was higher than 1. An ELISA to detect the IgG sub-classes was also performed for positive responders. Plates were coated with antigen, blocked and incubated with plasma diluted 1:100 as in the ELISA for total IgG. After washing, plates were incubated for 1 h at 22 °C with mouse mAbs to human IgG sub-classes diluted in blocking buffer according to the manufacturer’s specifications. The mAbs were from clones HP-6001 for IgG1, HP-6002 for IgG2, HP-6050 for IgG3, and HP-6023 for IgG4 (Sigma) and have been used previously to characterize IgG subclass reactivity. After incubation, plates were washed and incubated for 1 h at 22 °C with peroxidase-labelled goat anti-mouse antibody (KPL) diluted 1:1,000 in blocking buffer.

Plates were washed, incubated with ABTS (2,2′-Azinobis [3-ethylbenzothiazoline-6-sulfonic acid]-diammonium salt) substrate solution, and the (OD) measured as described above. Sub-class-specific prevalence for each antigen was determined from OD values using three standard deviation (SD) above the appropriate mean OD of 24 non-exposed controls as the cut-off for positivity. The corresponding cut-offs for PvAMA-1 were: IgG1 = 0.1910, IgG2 = 0.2401, IgG3 = 0.1558, IgG4 = 0.1828, and for PvMSP-1_19_ were: IgG1 = 0.1885, IgG2 = 0.2718, IgG3 = 0.1579, IgG4 = 0.1462.

### Statistical analysis

Epidemiological and experimental data were stored in Epi- Info 3.5.1 (CDC, Atlanta, GA, USA). Statistical analysis were performed using the R statistical environment and all p values were adjusted with false discovery rate method [37]. The risk of malaria associated with intestinal parasites was estimated using odds ratios (OR) and confidence interval of 95 % (CI). Chi-squared (χ^2^) with Yates correction was used to calculate differences in seroprevalence between groups. Wilcoxon-Mann–Whitney rank sum test was used for comparison between M and CI groups. Differences of reactivity index values were calculated with a pairwise test for multiple comparisons of mean rank sums (Nemenyi-Tests) [38]. P values of <0.05 were considered as significant.

## Results

### Prevalence of malaria and intestinal parasites

Combining the results across all tests (Table 1), 279 individuals were grouped according to their infection status: individuals infected with *Plasmodium* only (n = 16, M); individuals co-infected with *Plasmodium* and intestinal parasites (48, CI); individuals infected with intestinal parasites only (98, IP) and individuals negative (117, N). The group N was defined as subjects with no symptoms, negative for *Plasmodium* by thick blood smear and PCR and negative for intestinal parasites by direct wet mount microscopic and concentration techniques. CI and IP groups were composed of individuals positive for intestinal parasite infection: helminths only (H), protozoa only (P), and both helminths and protozoa (P+H).

**Table 1:** Distribution and number of individuals among the groups and subgroups according to malaria and intestinal parasites diagnosis

Groups	Subgroups	Description	Number of individuals	Total
Malaria (M)		Individuals infected with *Plasmodium* only	16	16
Co-infected (CI)	H	Individuals co-infected with *Plasmodium* and helminths only	3	
	P	Individuals co-infected with *Plasmodium* and protozoa only	39	48
	P+H	Individuals co-infected with *Plasmodium* and helminths + protozoa	6	
Intestinal parasites (IP)	H	Individuals infected with helminths only	17	
	P	Individuals infected with protozoa only	69	98
	P+H	Individuals infected with helminths and protozoa only	12	
Negative (N)		Individuals negative for malaria and intestinal parasites diagnosis	117	117
Total				279

*CI* subgroups, *H* individuals co-infected with helminths only, *P* protozoa only, *P* + *H* both helminths and protozoa

**Fig. 1:**
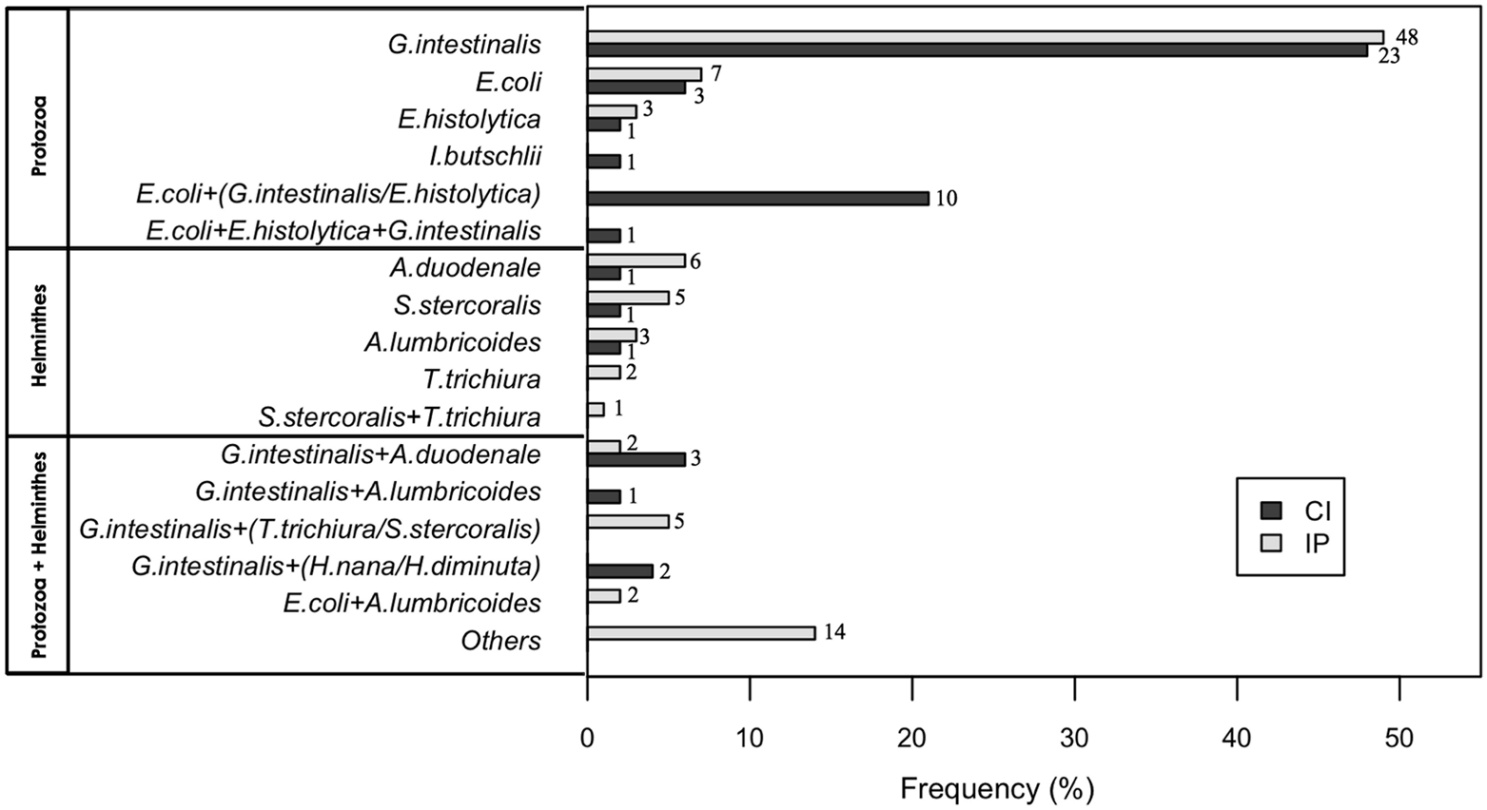
Prevalence of intestinal parasites among individuals co-infected with *Plasmodium* and intestinal parasites (CI) and individuals infected with intestinal parasites only (IP) in the studied population. *Black bars* indicate the frequency of intestinal parasite species in CI group and *grey bars* indicate the frequency of intestinal parasites in the IP group. Y axis illustrates the species corresponding to infections with Protozoa, Helminths and Protozoa + Helminths. *Numbers* on *top* of *bars* indicate number of individuals infected with each species of protozoa and helminths

In both M and CI groups, *P. vivax* was the most prevalent species (81.2 and 75.0 %, respectively). The prevalence of intestinal parasites was significantly higher in individuals infected with malaria (75 %) than with those who were not infected (45 %). Of note, all study participants with malaria presented clinical symptoms, such as history of fever and headache, and no difference was observed in the parasitaemia levels between malaria and co-infected groups. Among the 146 individuals infected with intestinal parasites, ten different parasite species were detected (Fig. 1), four intestinal protozoa (*Giardiaintestinalis*, *Entamoeba coli*, *Entamoeba histolytica*, *Iodamoeba butschlii*) and six helminths (*Ancylostoma duodenale*, *Strongyloides stercoralis*, *Ascaris lumbricoides*, *Trichuris trichiura*, *Hymenolepis nana*, *Hymenolepis diminuta*). *Giardia intestinalis* and *Entamoeba coli* were the most prevalent protozoa, found in single infections or associated with other species of protozoa and helminths (*Ancylostoma duodenale*, *Strongyloides stercoralis*). The prevalence of protozoan in CI group (81.25 %) was not statistically different from IP (70.4 %). In both, CI and IP groups, *Giardia intestinalis* and *Entamoeba coli* were the most prevalent protozoan and *Ancylostoma duodenale*, *Ascaris lumbrocoides* the most prevalent helminths.

### Characteristics of the studied groups

Table 2 summarizes the characteristics of the studied groups. Male were overrepresented in the M and CI groups and no differences were observed in the median age between all groups. Comparing M and CI groups, there were no differences in age, time since last malaria episode (LME) and eosinophils count. Anaemia was more prevalent in individuals from malaria and co-infected groups than in IP and N. Additionally, IP and N groups were similar and differed from M and CI in LME, and both groups presented higher eosinophils counts.

**Table 2:** Characteristic of the studied groups

	Malaria (+) N = 64		Malaria (−) N = 215	
	Malaria (M)^a^ N = 16	Co-infected (CI)^b^ N = 48	Intestinal parasite (IP)^c^ N = 98	Negative (N)^d^ N = 117
Gender N (%)				
M	11 (69)	34 (71)	49 (50)	55 (47)
F	5 (31)	14 (29)	49 (50)	62 (53)
Age (years)	24 (21–33)	31 (22–41)	30 (14–43)	31 (15–39)
Parasitaemia (parasites/µL)	2740 (738.5–56)	1816 (641–57)	(–)	(–)
LME (months)	6 (0–66)	3 (0–16)	24 (6–60)	10.5 (1–36)
Anemia (%)	56.2	43.7	*28* ^a*^	*23* ^a*b*^
Eosinophils (cells/mm^3^)	73 (36.75–138.75)	104 (42.5–328.5)	*328* (*185*–*72*)^a***b***^	*224 *(*146*–*44*)^a**b*^

All the values in the table represent the median (25–75 %) of the data

Differences between groups were calculated from pairwise test for multiple comparisons of mean rank sums (Nemenyi-Tests)

Differences of parasitaemia between co-infected and malaria group were calculated using Wilcoxon, a non-parametric t test

*LME* time since last malaria episode

Statistical differences of epidemiological parameters were expressed as * P < 0.05, ** P < 0.001, *** P < 0.0001

^a^Differences between indicated group and M group

^b^Differences between indicated group and CI group

### Specific IgG antibody responses to *Plasmodium vivax* AMA-1 and MSP-1_19_

**Fig. 2:**
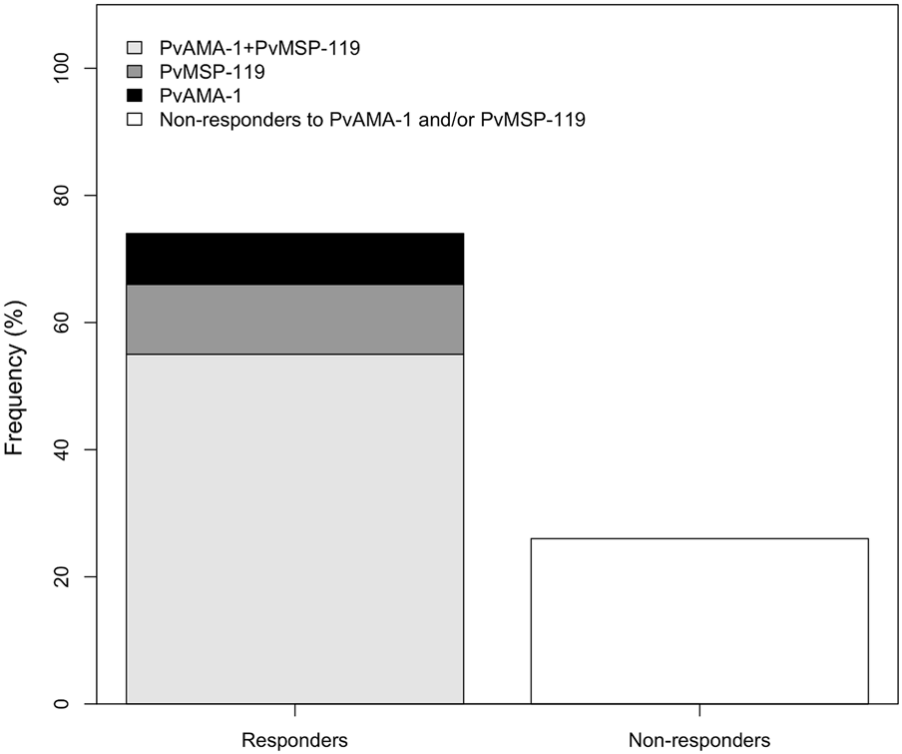
Frequency of specific antibody response to PvMSP-1_19_ and PvAMA-1 of individuals from a malaria-endemic area, determined by ELISA. Individuals were grouped in responders and non-responders for the recombinant proteins. The Y axis represents the frequency (%) of individuals responding to PvAMA1+PvMSP-1_19_, PvAMA-1 or PvMSP-1_19_ only, and individuals non-responders to PvAMA-1 and/or PvMSP-1_19_

**Fig. 3:**
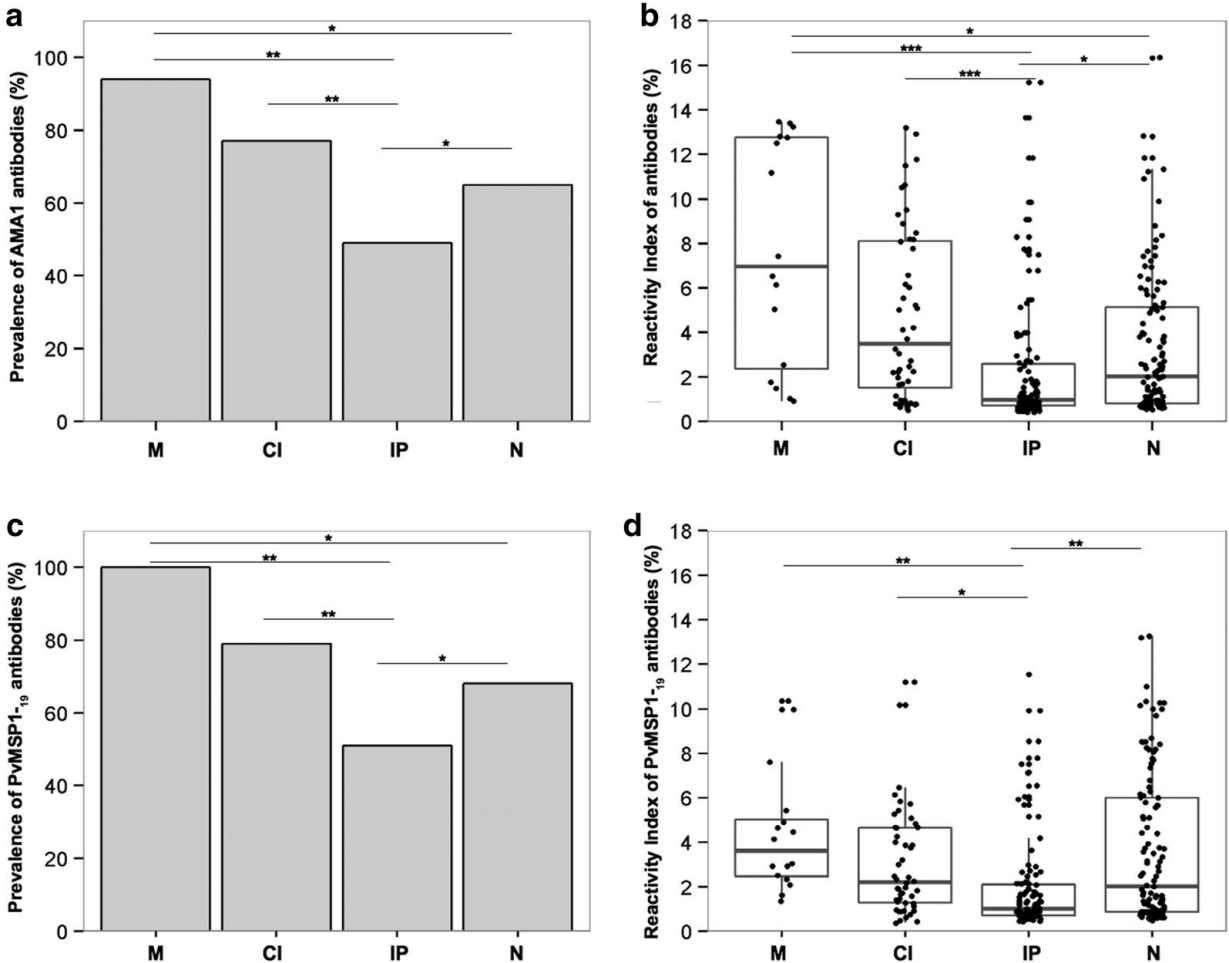
Prevalence and reactivity index of IgG antibodies to *P. vivax* AMA1 and MSP-1_19_ recombinant antigens. *M* individuals infected with *Plasmodium* only, *CI* individuals co-infected with *Plasmodium* and intestinal parasites, *IP* individuals infected with intestinal parasites only and *N* negative for both malaria and intestinal parasites. **a** Prevalence of PvAMA-1 IgG in M, CI, IP and N groups. **b** IgG Reactivity Index for PvAMA-1 among M, CI, IP and N groups. **c** Prevalence of PvMSP-1_19_ IgG in M, CI, IP and N groups. **d** IgG Reactivity Index for PvMSP-1_19_ among M, CI, IP and N groups. In **b** and **d** *panels*, the *horizontal bolded-bar* in the *Box* and *whisker* plot represents the median value and all individual data points are shown as *dots*. Whiskers extend ×1.5 of the interquartile range or to the minimum/maximum value, when these fall within ×1.5 of the interquartile range. Differences of reactivity index values were calculated from pairwise test for multiple comparisons of mean rank sums (Nemenyi-Tests) and all differences of prevalence between groups were calculated using X^2^ with Yates correction. Significant statistical differences are represented in the *bars* and the level of significance expressed as *P < 0.05, **P < 0.001, ***P < 0.0001

The percentage of individuals containing naturally acquired IgG antibodies against PvAMA-1 and PvMSP-1_19_ is presented in Fig. 2. IgG antibodies to PvAMA-1 and/or PvMSP-1_19_ were detected in 74 % of the population. The prevalence of individuals that recognize both proteins (55 %) was higher than those that recognize a single protein (8 % PvAMA-1 and 11 % PvMSP-1_19_). To determine whether the presence of co-infection was associated with impaired IgG responses, the prevalence of specific IgG directed to PvAMA-1 and PvMSP-1_19_ between groups were compared (Fig. 3). The prevalence of specific IgG was similar for both proteins in all four groups and among the groups, the lowest prevalence was in IP. The M group presented the highest frequency of IgG responders as compared to uninfected, and no appreciable differences were observed between M and CI groups (Fig. 3a, c). When plasma levels from individual serum samples were compared, the RI values obtained for the recombinant protein PvAMA-1 were not significantly higher than the values obtained for PvMSP-1_19_ (Fig. 3b, d). However, the RIs were lower in IP group for both proteins.

**Fig. 4:**
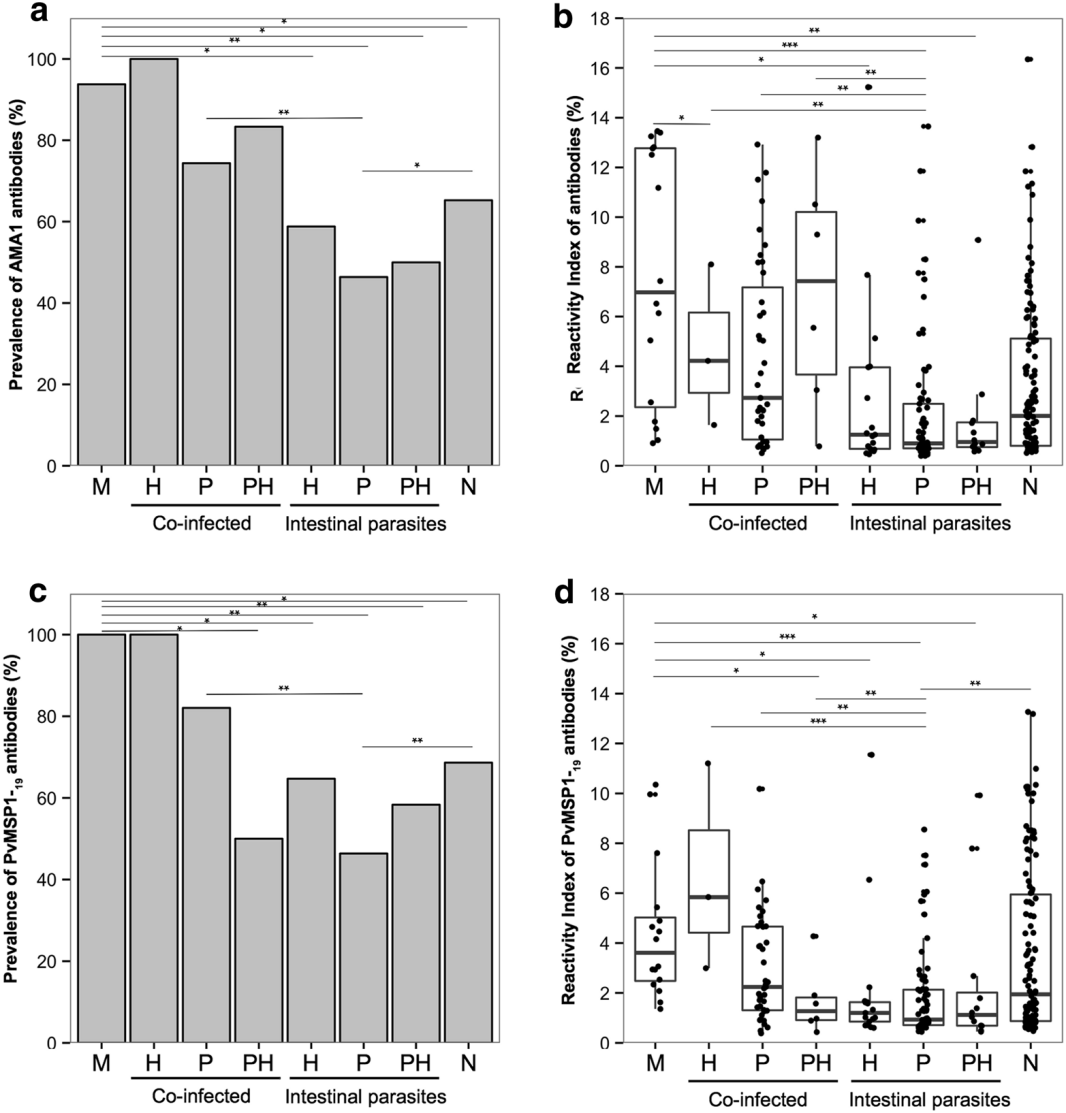
Prevalence and reactivity index of IgG antibodies to PvAMA-1 and PvMSP-1_19_ in the group of individuals co-infected with malaria and intestinal parasites (CI) and infected with intestinal parasites only (IP) according to intestinal parasite infection. *H* individuals infected with helminths, *P* individuals infected with protozoa, *PH* individuals infected with protozoa + helminths. **a** Prevalence of PvAMA-1 IgG in CI and IP groups among H, P and PH infected individuals; **b** IgG reactivity index for PvAMA-1 in CI and IP groups among H, P and PH infected individuals; **c** Prevalence of PvMSP-1_19_ IgG in CI and IP groups among H, P and PH infected individuals; **d** IgG Reactivity Index for PvMSP-1_19_ in CI and IP groups among H, P and PH infected individuals. In **b** and **d** *panels*, the *horizontal bolded-bar* in the *Box* and *whisker* plot represents the median value and all individual data points are shown as *dots*. Whiskers extend ×1.5 of the interquartile range or to the minimum/maximum value, when these fall within ×1.5 of the interquartile range. Differences of reactivity index values were calculated from pairwise test for multiple comparisons of mean rank sums (Nemenyi-Tests) and all differences of prevalence between groups were calculated using X^2^ with Yates correction. Significant statistical differences are represented in the *bars* and the level of significance expressed as *P < 0.05, **P < 0.001, ***P < 0.0001

Results in Fig. 4 show the prevalence and reactivity index of IgG response specific to PvAMA-1 and PvMSP-1 in the groups CI and IP among individuals infected with helminths (H), protozoa (P) and both protozoa and helminths (PH). There was no significant changes in the prevalence and RI of antibody to PvAMA-1 (Fig. 4a) and PvMSP-1 (Fig. 4b) in CI and IP groups when individuals infected with H, P and PH were compared in each group. However, IgG response to both PvAMA-1 and PvMSP-1 were lower in individuals with protozoa in the Intestinal parasites group when compared to Co-infected group. Similar results were also observed when the reactivity index were compared between the same groups. Although in some groups the sample size were small after stratification in H, P and HP, the group of individuals with P in both CI and IP groups seemed to be comparable. In the group Intestinal parasites, individuals infected with protozoa (P) also presented lower antibodies prevalence and RI to PvAMA-1 and PvMSP-1 than the group Malaria (p < 0.001 for PvAMA-1 and Pv-MSP-1) and negative (p < 0.01 for PvAMA-1 and PvMSP-1). In contrast, no differences were observed in prevalence and RI of antibodies to PvAMA-1 and PvMSP-1 between co-infected group infected with P and Malaria or Negative groups.

### Comparison of IgG subclasses to *Plasmodium vivax* PvAMA-1 and PvMSP-1_19_

**Fig. 5:**
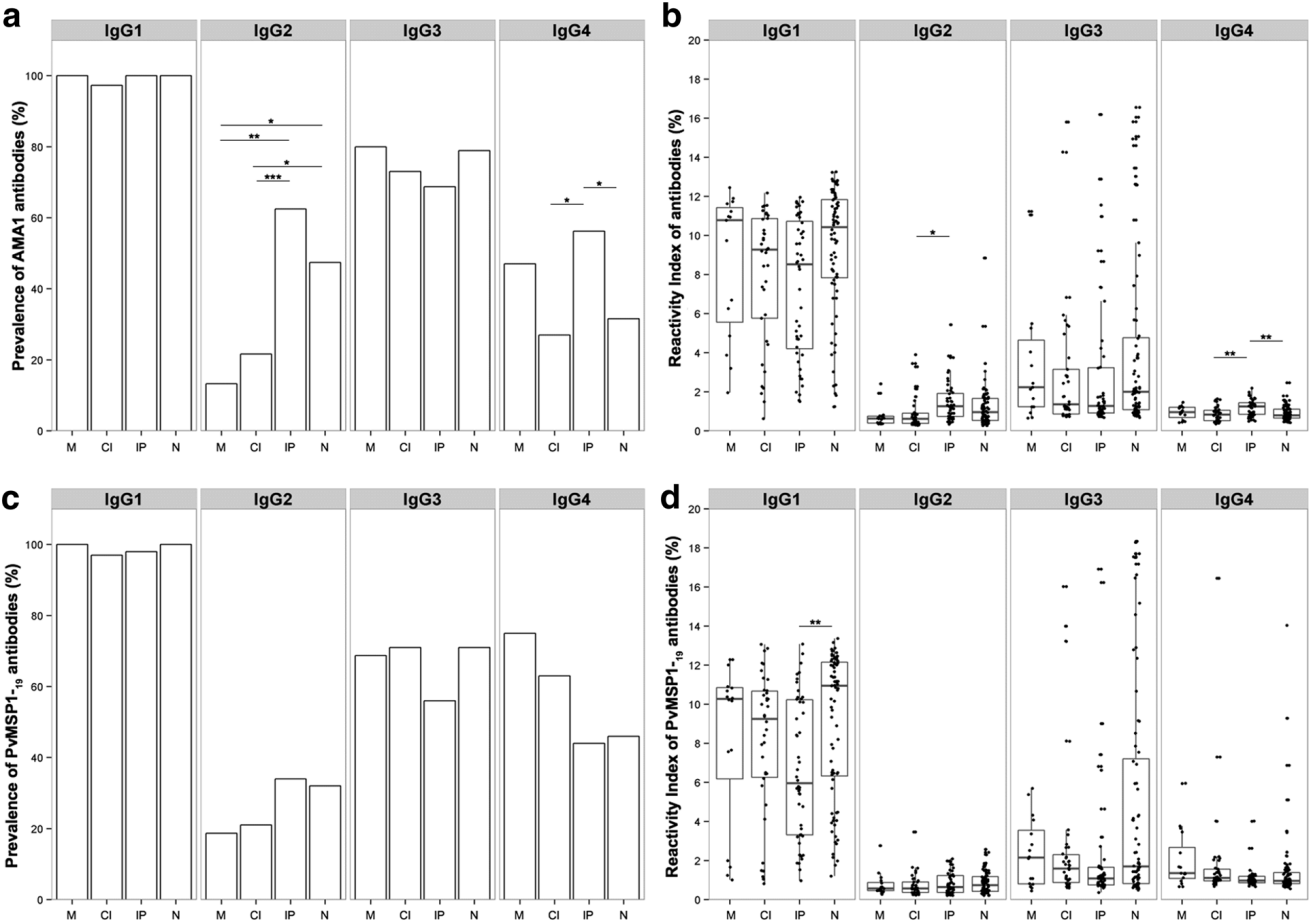
Prevalence and reactivity index of IgG subclasses to PvAMA-1 and PvMSP-1_19_. *M* individuals infected with *Plasmodium* only, *CI* individuals co-infected with *Plasmodium* and intestinal parasites, *IP* individuals infected with intestinal parasites only and *N* negative for both malaria and intestinal parasites. **a** Prevalence of IgG subclasses to PvAMA-1_19_ among groups; **b** IgG subclasses reactivity index for PvAMA-1 among; **c** prevalence of PvMSP-1_19_ IgG subclasses in M, CI, PI and N groups; **d** IgG subclasses reactivity index directed for PvMSP-1_19_ among M, CI, IP and N groups. X^2^ with Yates correction was used to evaluate subclasses differences into the groups. In **b** and **d** *panels*, the *horizontal bolded-bar* in the *Box* and *whisker* plot represents the median value and all individual data points are shown as *dots*. Whiskers extend ×1.5 of the interquartile range or to the minimum/maximum value, when these fall within ×1.5 of the interquartile range. Differences of reactivity index values were calculated from pairwise test for multiple comparisons of mean rank sums (Nemenyi-Tests) and all differences of prevalence between groups were calculated using X^2^ with Yates correction. Significant statistical differences are represented in the bars and the level of significance expressed as *P < 0.05, **P < 0.001, ***P < 0.0001

Since the IgG subclass produced in response to a given antigen may determine the function of the antibody, plasma samples positive for total anti-PvAMA-1 and anti-PvMSP-1 IgG were evaluated for IgG sub-class responses. Among the IgG responders, the cytophilic sub-class IgG1 and IgG3 were predominant in all groups for PvAMA-1 and IgG1, IgG3 and IgG4 for PvMSP-1_19_. In the case of non-cytophilic antibodies to PvAMA-1, IgG2 was significantly higher in IP and N group when compared to M and CI, while IgG4 was higher in IP group (Fig. 5a, c). No differences were observed in the prevalence of non-cytophilic antibodies specific to PvMSP-1_19_ among the groups. RIs of specific IgG sub-classes were also measured and increased IgG1 followed by IgG3 were detected for both proteins in all groups (Fig. 5b, d). In contrast, although IgG2 and IgG4 were frequent in the groups, their RI was low or similar for both proteins in all groups. However, RI of IgG2 to AMA-1 was significantly higher in IP when compared to CI group while IgG4 was higher in IP when compared to N and CI groups. Moreover, IgG1 RI to MSP-1 was significantly lower in IP group than in N group.

## Discussion

Although some studies have explored the influence of helminth co-infections on antibody production directed to *P. falciparum* antigens [21, 25, 39, 40], this is the first study to evaluate the influence of intestinal parasites on the acquired specific humoral immune responses to *P. vivax* malaria antigens [21, 24, 25, 39]. The present results show that in malaria-endemic area of Rondonia State, the prevalence of individuals singly infected with intestinal parasites (52.3 %) and co-infected with malaria and intestinal parasites (17.2 %) was higher than that singly infected with malaria (5.7 %). Some epidemiological studies have demonstrated an increased risk of infection by *P. falciparum* in those individuals co-infected with helminths but the results were conflicting [3, 6, 27, 40]. However, in the present study helminth infection was not predominant, the protozoan *Giardia intestinalis* alone or associated with other parasites was the most prevalent parasite in IP and CI groups, and *P. vivax* was the predominant *Plasmodium* species. Studies reporting the prevalence of co-infection in malaria-endemic areas of Brazil are scarce but a high prevalence of intestinal parasites has been reported in several areas of the Brazilian Amazon [27–29]. Although the prevalence of intestinal parasites was significantly higher in individuals infected with malaria, parasitaemia did not differ between co-infected and malaria-infected individuals. On the other hand, anaemia was frequent in both groups and increased numbers of eosinophils were observed in individuals with intestinal parasites and in the uninfected group. It seems that the haematological alteration observed in both malaria and co-infected groups is due to the effect of malaria, rather than intestinal parasites or co-infection. Indeed, anaemia is a common feature of acute malaria while increased number of eosinophils is common in helminths infections [41].

The study of antibody responses to *Plasmodium* antigens is a key process to the discovery and development of malaria vaccines. Several studies report high antibody response to *P. vivax* antigens in individuals exposed to malaria infection [42–44]. In malaria-endemic areas of Brazil, high prevalences of antibodies specific to *P. vivax* circumsporozoite protein [45], PvMSP-1 [46], MSP-9 [47], and PvAMA-1 [44] have been reported. In the present study, 73.8 % of individuals presented antibodies for at least one of the two studied proteins, indicating that both proteins are immunogenic. These values are comparable to other studies in the Amazon region [44, 46, 48, 49]. Higher prevalences and RI of specific antibodies were observed in the groups of individuals with patent *P. vivax* infection (groups M and CI) reaching almost 100 % in the group of individuals singly infected with malaria (M group). This confirms that PvAMA-1 and PvMSP-1_19_ are immunogenic molecules during natural malaria infections and that the presence of intestinal parasites does not interfere in the antibody response to both antigens. However, it cannot be ignored the reduced IgG prevalence and RI to both proteins in the group of individuals singly infected with intestinal parasites (IP), even when compared with the Negative Group. These results cannot be compared to those that reported reduced levels of IgG directed to *P. falciparum* antigens in helminths and *P. falciparum* co-infection, since helminth infections counted for only 19.7 % of the intestinal parasites detected in IP and 29.6 % in CI groups [21, 24]. On the other hand, Protozoa were the most prevalent intestinal parasites in the IP (70 %) and CI (80 %) and reduced antibodies prevalence and reactivity index in the IP group could be due to the presence of intestinal protozoa. However, in the group CI, the IgG prevalence and reactivity indexed were not reduced and were similar to the group infected with malaria only (M). In the CI group, the effect of intestinal protozoa might not be relevant when compared to the effect of *Plasmodium* in the immune response. It seems that *Plasmodium* have some effect on intestinal protozoa and not the contrary since lower antibodies response is only observed in IP group. Chronic protozoan infections have previously been suggested to be associated with type 1-regulatory immune response and combined with the induced pro-inflammatory response induced by the *Plasmodium* could balance the anti-inflammatory effect of the response [50]. Studies on malaria and protozoan co-infections are rare and the effect of intestinal protozoa on malaria infections or the contrary it is unknown.

Analysis of IgG isotypes response to PvAMA-1 and PvMSP-1_19_ antigens is important for evaluating protective activity as IgG subclasses differ in their immune effector functions and having such knowledge is important for understanding the immunity to vaccine development. The results of the present study confirmed studies that showed IgG1 and IgG3 isotypes, previously identified as protective to malaria, were the predominant subclasses in response to both antigens in all groups [44, 46, 51]. Therefore, the presence of intestinal parasites in malaria-infected individuals does not seem to alter the cytophilic and non-cytophilic response to PvAMA-1 and PvMSP-1_19_ in malaria and intestinal parasites co-infected individuals. However, in the IP group IgG1 reactivity index to MSP-1 were lower when compared to individuals from N. These results suggest that the presence of intestinal parasites can induce non-cytophilic antibodies that could counterbalance the production of cytophilic antibodies in IP group. The increased prevalence observed for non-cytophilic IgG2 response to PvAMA-1 in IP group does not seem to be due to intestinal parasites since N group also presented increased IgG2. In this group IgG4 RI to AMA-1 were also increased when compared to CI and N groups. Studies that investigate malaria and helminths co-infection reported a decrease of cytophilic IgG1 and IgG3 responses to MSP-1 and an increase in non-cytophilic IgG4 response to MSP-3 in individuals infected by *Ascaris lumbricoides*, *Strongyloides stercolaris*, *Trichuris trichiura* and *Hymenolepis nana* [52]. Similarly, a generalized decrease in cytophilic IgG directed to both GLURP, MSP-3, AMA-1, MSP-1, and MSP-2 and a significant negative association between *Schistosoma* infection and IgG1 and IgG3 directed to anti-malarial total extract was reported in individuals infected with *Schistosomahaematobium* and malaria [24]. In contrast, an increased IgG1 to MSP-1 response as well as IgG1 and IgG3 to total extract in malaria and *Schistosomahaematobium* co-infection was also reported [21]. Although protozoa infection was predominant in the present study, it is generally believed that in helminth infection the type 2 T helper (Th2) response induced by helminths could alter the natural immune response of the host to *Plasmodium*, due to the anti-inflammatory effect of cytokines induced by helminths. However, the cytokine profile of this population have been published and for malaria-infected individuals (M and CI groups) the profile showed high levels of IL-1, IL-6, TNF, IL-10, and CRP and decreased levels of IL-17A while for malaria-negative individuals (IP and N) the profile was high levels of IL-17A, NO and decreased levels of IL-10 and CRP [20]. Therefore, it seems that intestinal parasites co-infection (mainly protozoan) does not influence the plasmatic cytokine levels of acute malaria-infected individuals.

## Conclusions

The presence of antibody responses to both *P. vivax* AMA-1 and MSP-1 proteins in all groups indicated that the participants had been exposed to malaria infection and the IgG subclass responses were largely in agreement with previously published results. Although in the present work there were changes in total IgG directed to PvAMA1 and PvMSP-1_19_ in Intestinal parasites group, a decrease in IgG and in cytophilic responses associated to co-infections was not observed. These responses might perhaps relate to other factors such as antigen properties, number and time of exposure, host age and genetic determinants. Further studies should be conducted to determine the effect of intestinal protozoa in the immune response to malaria antigens.
